# The Fetal Allograft Revisited: Does the Study of an Ancient Invertebrate Species Shed Light on the Role of Natural Killer Cells at the Maternal-Fetal Interface?

**DOI:** 10.1155/2008/631920

**Published:** 2008-07-06

**Authors:** Amy Lightner, Danny J. Schust, Yi-Bin A. Chen, Breton F. Barrier

**Affiliations:** ^1^School of Medicine, Boston University, 715 Albany Street, Boston, MA 02118, USA; ^2^Department of Obstetrics, Gynecology and Women's Health, School of Medicine, University of Missouri-Columbia, Columbia, MO 65201, USA; ^3^Department of Medicine, Harvard Medical School, Massachusetts General Hospital, 55 Fruit Street, Boston, MA 02114, USA

## Abstract

Human pregnancy poses a fundamental immunological problem because the placenta and fetus are genetically different from the host mother. Classical transplantation theory has not provided a plausible solution to this problem. Study of naturally occurring allogeneic chimeras in the colonial marine invertebrate, Botryllus schlosseri, has yielded fresh insight into the primitive development of allorecognition, especially regarding the role of natural killer (NK) cells. Uterine NK cells have a unique phenotype that appears to parallel aspects of the NK-like cells in the allorecognition system of B. schlosseri. Most notably, both cell types recognize and reject “missing self” and both are involved in the generation of a common vascular system between two individuals. Chimeric combination in B. schlosseri results in vascular fusion between two individual colonies; uterine NK cells appear essential to the establishment of adequate maternal-fetal circulation. Since human uterine NK cells appear to de-emphasize primary immunological function, it is proposed that they may share the same evolutionary roots as the B. schlosseri allorecognition system rather than a primary origin in immunity.

## 1. THE HUMAN MATERNAL-FETAL RELATIONSHIP

Human pregnancy poses a fundamental immunological problem because the placenta and fetus are genetically different than the host mother. In 1953, Sir Peter Medawar posited the fetus as a successful solid organ transplant, initiating a wave of research that attempted to explain fetal implantation in terms of classical transplantation immunology [1]. The mediators of the adaptive immune system are thought to be responsible for transplant rejection. High intrinsic allogeneic interactions of CD8+ T cells with foreign major histocompatibility complex (MHC) class I molecules are considered to be the major mediator, but minor histocompatibility antigens, CD4 T cells, and specific antibodies also play a significant role. Accordingly, many have studied fetal survival in the context of escape from the maternal adaptive immune system via anatomical separation, antigenic immaturity of the fetus, and/or immunological inertness of the mother. However, mother and fetus are not truly anatomically separate because placental tissue, derived from fetal trophoblasts, invades deep into maternal uterine myometrium along spiral arteries, replacing maternal arterial endothelium. This allows for the exchange of cellular and noncellular material between mother and fetus. The presence of fetal blood cells, leukocytes, and trophoblast cells in the maternal circulation contradicts a role for the placenta as an effective immunologic barrier [2]. In addition, mothers do mount an immune response to paternal antigens and, not infrequently, possess specific antibodies or cytotoxic T cells against paternal MHC molecules. The presence of this response has been shown to have no effect on pregnancy outcome [2–4], an observation hypothesized in the past to be related to the presence of paternal-antigen specific maternal regulatory T-cells, of blocking antibodies that protect the fetus, and/or of immunosuppressive plasma proteins [2–6]. It is also doubtful that the uterus is an immunologically privileged site, because, unlike the brain or testes, it is endowed with a well-developed lymph drainage system [3]. Finally, while patients with autoimmune diseases provide ample evidence that there are maternal immunomodulatory changes during gestation, maternal immunity can certainly still mount a successful adaptive response against nonself.

Colonial ascidians, a class of marine invertebrates commonly known as sea squirts or tunicates, may offer a unique perspective on the maternal-fetal relationship. Like mother and fetus, colonial ascidians experience a natural exchange of allogeneic tissue. Colonies that are successful in this exchange somehow avoid mutual rejection and reap the benefits of chimeric life. This phylogenetically ancient species manages this balancing act without benefit of an adaptive immune system (which first evolved in cartilaginous fish) [4]. Study of the immunological mediators for this system of natural allorecognition may reveal the evolutionary precursor to modern mechanisms for the human parallel of colonial life: the maternal-fetal relationship. The colonial ascidian, B. schlosseri, is interesting for three reasons: (1) its colonial chimerism has been extensively studied, (2) it appears not to possess an adaptive immune system, and (3) it is of the urochordate subphylum, a group of chordates thought to lie on the evolutionary line leading directly to vertebrates.

## 2. BOTRYLLUS SCHLOSSERI

**Figure 1:**
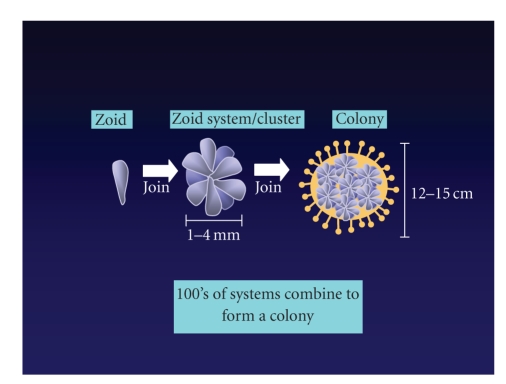
Botryllus schlosseri colony formation. The B. schlosseri individual is called a zooid. It attaches to the substrate that it will grow on by one end. Its free end has two openings or siphons. The zooid reproduces asexually and groups of 3–12 zooids will group together to form a cluster or system, with the free end of each zooid dumping into a central chamber. Clusters, in turn, join to form the B. schlosseri colony. All clusters in a colony are housed in a common tunic and share a common vasculature. Vessels in a colony terminate in peripheral ampullae that, while covered in the tunic, present sites of colony-to-colony interactions.

**Figure 2:**
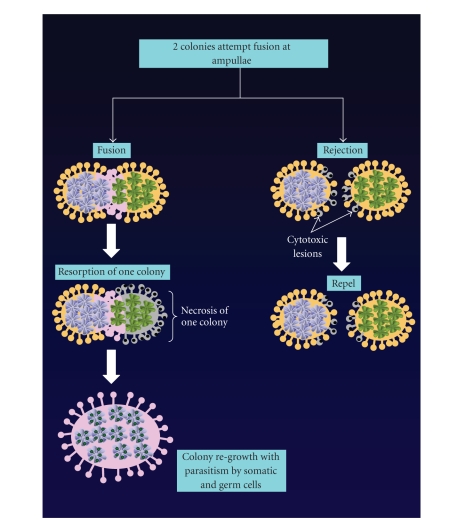
Botryllus schlosseri colony interactions. In the wild, colonies of B. schlosseri can become quite large, and genetic analyses reveal these colonies to be multichimeric. Interactions between genetically distinct colonies are governed by an allorecognition system controlled by the fusibility/histocompatibility locus (Fu/HC). When genetically distinct colonies contact one another, they will anastamose if one or both Fu/HC loci are recognized as self. Fusion involves the creation of a shared vasculature and common tunic. If neither Fu/HC loci are shared, rejection will occur and inflammatory lesions will develop at sites of contact. One colony will be largely resorbed after acceptable recognition and successful colony fusion, although some germ and somatic cells of the resorbed individual will persist in the survivor, creating a parasitic relationship.

Recent reviews have summarized the life cycle and allorecognition of B. schlosseri in detail, but a brief summary is presented here [5, 6]. B. schlosseri, commonly called the star ascidian or golden star tunicate, is native to Europe, but has spread to coastlines around North America, Asia, and Australasia. Each individual colony member (zooid) has its ventral side attached to a substrate, upon which the colony grows laterally, while the free dorsal end has two openings, the buccal and atrial siphons (Figure 1). Zooids measure 1–1.5 millimeters in length. They are usually grouped together into star-shaped clusters or systems of 3–12 members, with the atrial siphons of all individuals opening into a common chamber in the cluster's center. Colonies consist of a number of clusters embedded in a common gelatinous matrix, the tunic. The Botryllus colony has the potential to grow quite large and may be comprised of up to a thousand individuals [7]. Zooid propagation occurs through asexual reproduction, so all zooids within an individual colony are genetically identical. A common vascular system connects each zooid cluster and terminates in peripheral sacs called ampullae that are not only important for colony adhesion to substrate, but also allow for intercellular contact and exchange among zooids. When the peripheral ampullae of different colonies come into contact, they either anastomose and remodel their vessels to form a shared vasculature under a common tunic [8], or develop inflammatory lesions, indicating a site of rejection (Figure 2) [9, 10].

This natural chimerism is mediated by an allorecognition system, primarily controlled by a single, highly polymorphic locus, the fusibility/histocompatibility locus (Fu/HC) that contains up to several hundred codominantly expressed alleles [11, 12]. B. schlosseri allograft reactions differ from MHC-dependent vertebrate graft rejection. Whereas mammalian solid organ transplant rejection is thought to rely on host T-cell engagement with foreign MHC molecules (nonself), B. schlosseri rejection is based on failure to recognize self as encoded by the Fu/HC locus. Rejection occurs only when no Fu/HC allele is shared between the two individual colonies. Fusion via a shared vasculature is permitted when at least one Fu/HC allele is shared. In contrast, vertebrate graft rejection occurs if either MHC haplotype differs. Lastly, and unlike vertebrate MHC molecules, there is no evidence that the Fu/HC system functions in antigen presentation, although it may encode genes involved in innate immunity, such as complement and pattern recognition receptors [13, 14].

B. schlosseri colony fusion in the presence of acceptable allorecognition may have several advantages. Larger size may aid in the control of a long-term feeding substrate and may limit inter-specific competition. In fact, the respiration rate of individual zooids decreases as colony size increases [15]. It has been proposed that environmental conditions (primarily water flow and food supply) dictate the number of zooids per generation, the number of coexisting generations in a colony [16], and the rate of colony resorption (see below). While it is still unclear if colony forming is a true survival technique, it stands to reason that colony fusion shifts the unit upon which natural selection acts from the individual's genotype to that of the fused colony. The high degree of somatic “heterozygosity” in a multichimera may protect it from hierarchal somatic resorption and thereby create the best somatic “container” for the reproduction of its gametes.

Whereas the formation of multichimeras seems to be preferred in nature, as evidenced by the preference of larvae to settle in aggregates of kin colonies [13] and to co-settle with parental colonies [14], phenotypic resorption of individual colonies occurs in the majority of typically bichimeric animals that thrive in the laboratory setting. In most of these bichimeric colonies, this resorption after initial fusion follows a strict genetic hierarchy. (if colony A resorbs colony B and colony B resorbs colony C, then colony A will always resorb colony C.) Heterozygotes at the Fu/HC locus are always dominant in that they always resorb homozygotes [17]. Two additional genes, referred to as the resorption/histocompatibility (Re/HC) loci, also determine the hierarchy of the resorption reaction [18]. These genes are not linked to the Fu/HC locus and appear to sort independently.

To understand the molecular basis of allorecognition, chimeras were designed from two colonies, each heterozygous at the Fu/HC locus, and sharing one allele [19]. These investigations demonstrated that the mediators and effectors of histoincompatibility circulate throughout the common vascular system of fused colonies and that blood components of even the resorbed genotype remain following colony resorption. In B. schlosseri, hemocytes differentiate along two defined pathways, becoming either (1) phagocytes that are macrophage—like in their immunostaining characteristics or (2) cytotoxic cells, including hyaline amebocytes [20] and morula cells [21, 22]. Of these cell types, the morula cells appear to be the major immunomodulatory hemocytes in ascidians as they alone display significant changes in cytokine (IL-1*α* and TNF-*α*) secretion upon immunostimulation [23]. Morula cells are also the most viable candidate mediators of the alloimmune-mediated resorption [22, 24]. These hemocyte-derived ascidian blood cells are capable of cytotoxic activity, they proliferate in response to T-cell and B-cell mitogens, and they accumulate at the ampullae during rejection reactions [25]. Ascidian blood cells and human lymphocytes share common immunoglobulin superfamily proteins and a highly polymorphic disulfide linked, membrane bound heterodimer that may be a precursor to the MHC products [26, 27].

## 3. CELLULAR PARASITISM

Several studies have demonstrated that after allogenic fusion and subsequent colony resorption, germ and somatic stem cells of the resorbed partner can survive and replicate within the other partner's soma and/or gonads (Figure 2) [28]. Thus, the initial fusion/resorption reaction may actually result in somatic or germ cell parasitism at the cellular level [29]. However, if all individuals could fuse, one supercompetitor genotype could dominate and homogenize the genome of the entire species [30]. Therefore, while the initial fusion/rejection self-recognition reaction likely evolved to facilitate competition among closely related kin, the rejection prevents one dominant genotype from homogenizing the genotype of the entire species through germ-cell parasitism, with the benefit of preserving genetic diversity.

In addition, it appears that somatic and germ cell resorption events are independent of one another [31]. The genotype selected somatically may not be the one selected gametically. The apparent somatic “winner” by gross resorption could turn out to be the gametic “loser” if all of its germ cells are colonized and parasitized by the resorbed individual. In fact, both types of cellular parasitism seem to represent independent stem cell lineages existing simultaneously [14]. The retention of stem cells from seemingly resorbed individuals allows the colony to be phenotypically plastic in response to its environment. Indeed, evidence has shown that environmental changes can alter the colony phenotype [9].

## 4. B. SCHLOSSERI—THE EVOLUTIONARY LINK

Based on the chordate characteristics of its larva, the ascidians appear to be among our closest invertebrate relatives [32]. If B. schlosseri shares a common invertebrate ancestor with humans, it must sit at a critical point in vertebrate evolution, just before the development of the adaptive immune system. There are, in fact, examples of invertebrate species, such as the marine organism Hydractinia, that predate B. schlosseri by several hundred million years, but exhibit a highly specific allorecognition system [33]. Recognition in these organisms, however, appears to occur via interactions in ectodermal- and/or endodermally-derived cells [33–35].

Natural chimeric relationships, which are integral to the B. schlosseri life cycle, exist rarely among eutherians—organisms utilizing a placenta to allow *in corpus* fetal development. Examples include spontaneous early fusion of dyzygotic twins, rare cases of germ cell chimerism, and proliferation of fetal cells in the maternal blood system after parturition [36, 37]. One instance of natural chimerism, however, is remarkably common—the maternal-fetal allograft itself. Evolutionary remnants of an allorecognition system found in the common ancestor shared by B. schlosseri and modern eutherians may govern maternal-fetal allogeneic interactions. For B. schlosseri, a single self-identification system appears to govern its initial allogeneic interactions. In higher vertebrates, inhibition of NK cell cytolysis by self-MHC class I molecules resembles the Fu/HC system of B. schlosseri in that it is based on the suppression of cytolytic activity via recognition of self.

## 5. NK CELLS

It is commonly accepted that NK cells share a developmental precursor with T lymphocytes [38]. In fact, some believe that the NK cells, found in all invertebrates, are the evolutionary precursor to lymphocytes [38, 39]. NK cells are a component of the innate immune system, thought to be important in the immune response against viruses and tumors that may downregulate expression of classical MHC molecules [38–40]. They are non-T, non-B lymphocytes which do not require prior exposure to act and do not act by recognition of nonself. NK cell activation is regulated by a complex interplay between multiple activating and inhibiting signals [41]. Inhibitory signals are mainly delivered by the self MHC class I products normally present on the surface of nucleated cells. The activating signals may be delivered by several recognized cell surface receptors present on NK cells, including CD16, the killer inhibitory receptor (KIR)2DS, and NKp46 [42, 43]. NK cells kill target cells via discharge of cytoplasmic granules or by cytokine signaling to nearby macrophages.

Seventy percent of human endometrial leukocytes during early pregnancy are large granulated lymphocytes, while 20% are macrophages, and 10% are T cells. B cells and peripheral CD16+CD56+NK cells are nearly absent [44]. CD16 is a transmembrane receptor for immunoglobulin (Ig)G and CD56 is an Ig family glycoprotein expressed on the surface of all NK cells. Granulated lymphocytes had been identified in the endometrium of many species, but they were not identified as NK cells until intense expression of CD56 was demonstrated. Note that the CD56 marker is only found in higher order primates and not found in the species that had been studied prior to its discovery (e.g., rodent, porcine, and bovine) [45].

## 6. UTERINE NK CELLS

In humans, uterine granulated lymphocytes differ immunophenotypically from peripheral blood NK cells in that they are CD56^bright^ and CD16−, whereas peripheral blood NK cells are CD56^dim^ and CD16+ [39, 46]. In addition, although reverse transcriptase polymerase chain reaction (RT-PCR) has confirmed that uNK cells express the same families of inhibitory and activating receptors as peripheral NK cells, they do so in different proportions, and with variability among individual women [39, 46, 47].

Uterine NK cells are only found in the human endometrium during the menstrual years, implying an active role in reproduction. Their population peaks during the progesterone-dependent secretory phase of the menstrual cycle. After implantation, they persist in the uterine mucosa, gathering in high concentrations at the site of placental invasion. Although substantial numbers of CD56+ cells remain in placental tissue during the third trimester of pregnancy [48], their number gradually decreases over pregnancy and they are usually absent in term decidua [39, 49].

The uterine mucosa is the only tissue in the human body where NK cells of any kind are known to proliferate and survive in large numbers. In the late secretory phase and early pregnancy, decidual uNK cells appear localized to the stratum functionalis, forming aggregates around spiral arteries and glands [44, 46]. Initially, it was speculated that this localization was due to the effect of the diffusion of progesterone from blood into the perivascular tissues, since large numbers of uNK cells are seen after progesterone treatment. However, more recent evidence suggests the perivascular distribution reflects the trafficking of uNK precursors from the circulation [46, 50].

## 7. TROPHOBLAST MHC MOLECULES

**Figure 3:**
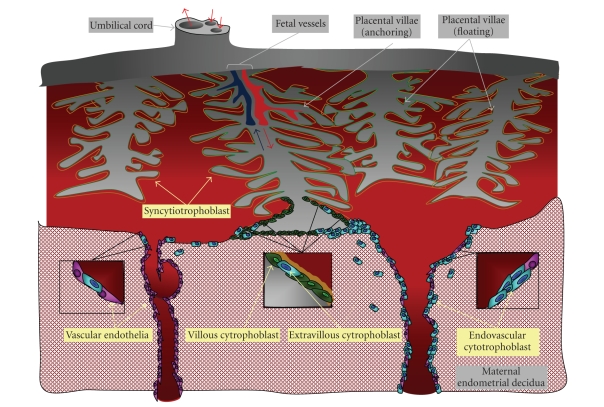
The human maternal-fetal interface. Fetal blood enters and exits the placenta via the umbilical cord. Fetal vessels lie at the core of each placental villae and all villae are lined externally by trophoblast cells. The cells of the inner layer (green) are called villous cytotrophoblast cells. Those of the outer layer develop from the villous cytotrophoblast through syncytialization and are called syncytiotrophoblast (orange). Floating villae are completely bathed in maternal blood; anchoring villae traverse the intervening blood-filled space to attach to the maternal decidua. At the tips of the anchoring villae, a subpopulation of cytotrophoblast cells leaves the villae to invade the maternal endometrial decidua. Here, these extravillous cytotrophoblast (blue) cells encounter populating maternal immune cells. Some extravillous cytotrophoblast cells will invade the maternal uterine arteries and become endovascular trophoblast cells (blue). Appropriate vascular invasion by endovascular trophoblast cells results in remodeling of the uterine vasculature (inset, right) and adequate placental perfusion. Poor vascular invasion by endovascular trophoblast cells results in limited remodeling of the uterine vasculature (inset, left), a condition associated with adverse pregnancy outcomes.

Placenta and uterine mucosa are the primary tissues of the maternal/fetal interface. Placental villous syncytiotrophoblast and extravillous cytotrophoblast cells are the fetal cells most intimately contacted by maternal cells (Figure 3). According to mRNA analysis, these cells do not express normal levels of classical MHC I or MHC II molecules [51, 52]. Instead, the extravillous cytotrophoblast cells express two nonclassical MHC I molecules, human leukocyte antigen (HLA)-G and HLA-E, and low levels of the classical MHC I molecule, HLA-C [53, 54]. When compared to HLA-A and HLA-B, HLA-C, -E, and -G exhibit much lower surface expression, fewer alleles, reduced polymorphism at peptide binding sites, and lower intrinsic reactivity with CD8+ T-cells [53, 55]. This suggests that the primary function of trophoblast HLA molecules is something other than the presentation of foreign peptide to CD8+ T-cells. In addition, trophoblast MHC products are uniquely resistant to the strategies that multiple viral pathogens employ to evade immune detection [53, 56–58], suggesting that these class I products do not have a primary role in pathogen defense.

Substantial evidence demonstrates that MHC molecules, including trophoblast MHC products, play a role in the inhibition of NK cell mediated target lysis. Inhibitory receptors on NK cells include members with immunoglobulin-like characteristics [leukocyte Ig-like receptors (LIRs) and killer Ig-like receptors (KIRs)] and heterodimers with characteristics of C-type lectins (CD94/NKGs) [59]. It is now known that the KIRs recognize the classical MHC products (HLA-A,-B and -C), and CD94/NKG2 recognizes HLA-E molecules [46, 60, 61]. The recent discovery of the crystal structure of HLA-G [62] supports tight binding to LIR1 and LIR2 and poses a plausible ligand-receptor interaction. Others have demonstrated interactions between HLA-G and KIR2DL4 as well [63]. Furthermore, treatment of fetal cells with monoclonal antibodies directed against HLA-G and HLA-C abolishes their resistance to NK cell mediated lysis [64], while transfection of HLA-G into NK susceptible cell lines prevents NK cytolysis [65]. Although HLA-G expression has been recently described in multiple malignancies and chronic inflammatory conditions, it is never expressed in significant amounts in normal tissues other than fetal trophoblasts [66].

## 8. UNK CELL FUNCTION

The most important role of uNK cells may be the regulation of fetal implantation and initial establishment of the placenta through vascular remodeling. During implantation, fetal trophoblast tissue invades muscular uterine tissues and spiral arteries, converting the latter into enlarged structures with limited vascular resistence, poor responsivity to vasoactive signals, and the capability for high conductance (Figure 3). These changes are necessary to supply sufficient blood volume to the fetus. When trophoblast invasion is not sufficient, fetal circulation is compromised resulting in miscarriage, idiopathic uterine growth retardation (IUGR), stillbirth, and pre-eclampsia [55, 66]. In fact, decreased expression of HLA-G on human extravillous trophoblast is associated with reductions in placental invasion, shallow placentation, and development of pre-eclampsia, a maternal disease associated with impaired placental vascularization and development [67]. Recognition of trophoblast MHC products by uNK cells could serve as one trigger for cytokine release to induce and modulate this vascular remodeling. The repertoire of cytokines secreted by uNK cells includes VEGF-C, PIGF, Ang-2, TNF-alpha, IL-10, GM-CSF, IL-1Beta, TGF-Beta1, CSF-1, LIF, and IFN-gamma [68–71], any or all of which may help to establish a functionally healthy maternal-fetal circulation [39, 47]. One cytokine, IFN-gamma, seems to be particularly involved in the control of trophoblast invasion and the remodeling of uterine spiral arteries during the first half of pregnancy. Indeed mice deficient in either uNK cells or IFN-gamma have implantation site abnormalities and failure of decidual artery remodeling. They exhibit fetal loss in the second half of gestation, an overall smaller placental size, and progressive lesions suggestive of hypertension-mediated arteriosclerosis in the uterine vessels [72]. These defects can be reversed by grafting bone marrow from NK cell competent, but T and B cell immunodeficient, severe combined immunodeficiency (SCID) mouse donors [73] or RAG-2-/-IFNgamma-/-mice treated with murine IFN-gamma [74]

A more precise understanding of the roles of uNK cells in human pregnancy awaits future study of uNK cell receptor expression and cytokine release. Studies such as in vitro trophoblast invasion assays in the presence of Ang-2 [75], TNF-alpha [76, 77], IFN-gamma [77], and TGF-beta1 [78, 79] have provided insight into the role of cytokine production in the control of extravillous trophoblast invasion. Similar in vivo study of cytokine production and effects at the maternal-fetal interface is highly complicated due to the short half life of cytokines, difficulty in staining for endogenous versus exogenous cytokines, considerable variation in the microenvironment of the uNK cell, and obvious ethical considerations.

## 9. EVOLUTIONARY CONSIDERATIONS

We have presented evidence that mammalian uNK cells are important for the development of the placenta's vascular supply: immunotropism appears to both promote trophoblast invasion and the remodeling of spiral arteries. Vascular fusion among terminal ampulla after acceptable Fu/HC-mediated recognition in B. schlosseri chimerae leads to allogenic interactions reminiscent of those established at the maternal/fetal interface. Although the morula cells that mediate the recognition/rejection response of B. schlosseri can help in the foreign cell recognition that determines the activity of phagocytic cells [80, 81], they are one of only 3 types of hematocyte-derived cells in ascidians. The latter, recently-described function is partially redundant to the role of primary phagocytic cells and may have been secondarily gained through adaptation. Like human uNK cells, morula cells are thought to rely largely on the recognition of self rather than the engagement of nonself ligands. It is from this comparison that we hypothesize that allorecognition and innate immunity are largely distinct entities with separate evolutionary origins.

Evolutionary precursors of fetal trophoblasts may have expressed trophoblast MHC products to engage uNK cells, and only later evolved the classical MHC molecules that participate in immune defense. It is not difficult to imagine that peripheral NK cells evolved from uNK or a common precursor cell, possibly in response to viral pathogens not effectively curtailed by MHC-T-cell-based immunity. One might then speculate that the intrinsic reactivity existing between T cells and foreign MHC has its origin in NK cell-trophoblast MHC allorecognition.

Can a system of allorecognition used in modern sessile marine invertebrates for promoting survival and reproduction have given rise to the vertebrate adaptive immune system? Clearly, given the survival of invertebrates, all of whom possess only innate immunity, adaptive immunity is not essential to survival and proliferation of some species. Intrinsic allorecognition, while present in both colonial ascidians and mammals, serves different, but vital, functions in each case. To further unravel the mystery of the fetal-maternal relationship, the uNK cell lineage needs to be traced back in evolution. Much more information will become available once the genetic analyses of human uNK cells and the B. schlosseri NK-like cells are performed. Thus far, multiple analogs and several true homologues linked to MHC genes, cytokines, signaling proteins, and adhesion molecules have been found in the genome of B. schlosseri. Recently, it was shown that monoclonal antibodies directed against human IL-1 and TNF-alpha could stain a subpopulation of stimulated B. schlosseri blood cells [23]. However, no classic MHC molecule or HLA-G homolog has yet been found in the B. schlosseri genome [14], and few Fu/HC gene products have been identified [82].

HLA-G-like genes are present in lower primates [83–85]. In fact, an HLA-G-like product is the only class I molecule expressed in certain New World Monkeys. This demonstrates that this molecule can function effectively in innate and adaptive immunity. Further, significant polymorphism is detectable in the NK receptor-, T cell receptor-, and peptide-binding regions of the HLA-G-like molecules of New World Primates, but not of Old World Monkeys, suggesting divergent evolution and divergent functions [84]. The former certainly has primary immune function and a role in pathogen defense; this is unlikely to be true for the latter. The presence of trophoblast MHC homologues should be further investigated in lower mammals and genetic linkage studies should be performed with the Fu/HC locus to determine a possible relationship. The most obvious obstacle to finding a true evolutionary relationship is that homologous molecules operating in nonidentical systems may have different constraints on structural conservation and, thus, may display distinct patterns of change [30]. Still, whether B. schlosseri allorecognition eventually proves to be truly homologous or merely analogous, its use for comparison has resulted in fresh insight into the governing mechanisms and the evolutionary origins of the human maternal-fetal relationship.

## LIST OF ABBREVIATIONS:

CD: Cluster of differentiation
Fu/HC: Fusibility/histocompatibility
HLA: Human leukocyte antigen
Ig: Immunoglobulin
KIR: Killer immunoglobulin-like receptor
LIR: Leukocyte immunoglobulin-like receptor
MHC: Major histocompatibility complex
NK: Natural killer
Re/HC: Resorption/histocompatibility
RT-PCR: Reverse transcriptase polymerase chain reaction
SCID: Severe combined immunodeficiency
uNK: Uterine natural killer
